# The complete chloroplast genome of *Mitreola yangchunensis* (Loganiaceae)

**DOI:** 10.1080/23802359.2020.1835575

**Published:** 2020-11-20

**Authors:** Xiaolang Du, Lan Cao, Shasha Sheng, Guoyue Zhong, Zejing Mu

**Affiliations:** Research Center for Traditional Chinese Medicine Resources and Ethnic Minority Medicine, Jiangxi university of Traditional Chinese Medicine, Nanchang, China

**Keywords:** Complete chloroplast genome, *Mitreola yangchunensis*, Gentianales

## Abstract

The complete chloroplast (cp) genome of *Mitreola yangchunensis* was sequenced and assembled for the first time. The genome is 154,665 bp in length, containing a large single-copy (LSC) region of 85,351 bp, a small single-copy region (SSC) of 18,218 bp and a pair of inverted repeats (IRs) of 25,548 bp. It contains 113 unique genes, including 79 protein-coding genes, 30 tRNA genes, and 4 rRNA genes. The overall GC content is 37.9%, while the corresponding values of LSC, SSC, and IR regions are 35.9, 32.0, and 43.4%, respectively. Phylogenetic analyses using complete cp genomes showed that *M. yangchunensis* is most closely related to *Mitrasacme pygmaea* in Loganiaceae, and Gelsemiaceae and Loganiaceae form a single cluster with high support value.

*Mitreola* L. is a small genus of the family Loganiaceae with about 15 species, which is mainly distributed in China (Li and Leeuwenberg 1996; Wang 2018; Shan et al. 2019). Among them, *Mitreola yangchunensis* Q. X. Ma, H. G. Ye & F. W. Xing is endemic to Guangdong province, China (Ma et al. 2010). The morphological characteristics of *M*. *yangchunensis*, especially the leaves and stems, are similar to those of *M*. *reticulate* Tirel-Roudet. In this study, we sequenced and assembled the chloroplast (cp) genome of *M. yangchunensis* for the first time. It will provide an opportunity to examine morphological evolution and understand the phylogeny of *Mitreola*.

Fresh leaves of *M. yangchunensis* were collected from Yangchun city, Guangdong Province, China (N22°11′9.07″, E111°44′46.44″) and quickly dried with silica gel for DNA extraction. Herbarium voucher (Voucher No. 20180321) is stored at Medicinal Herbarium, Jiangxi University of Traditional Chinese Medicine, Nanchang, China. Total genomic DNA was extracted using the modified CTAB method (Doyle and Doyle 1987). Paired-end reads were generated on an Illumina NovaSeq platform with a read length of 150 bp. The GetOrganelle v1.6.2 (Jin et al. 2018) were used for the *de novo* assembly of cp genome. Genes were annotated by PGA (Qu et al. 2019) and visually checked in Geneious v8.0.2 (Kearse et al. 2012) using the cp genome of *Catharanthus roseus* (GenBank accession NC_021423) as reference. The predicted transfer RNAs (tRNAs) were confirmed by tRNAscan-SE 2.0 (Lowe and Chan 2016). The final complete cp genome sequence of *M. yangchunensis* has been submitted to GenBank under the accession number MT471262. Raw reads were deposited in the GenBank Sequence Read Archive (SRA SRR12719804).

The complete cp genome of *M. yangchunensis* is 154,665 bp in length with high coverage (mean 4017×). It has a typical quadripartite structure, including a large single-copy (LSC) region of 85,351 bp, a small single-copy region (SSC) of 18,218 bp, and a pair of inverted repeats (IRs) of 25,548 bp. There are 79 protein-coding genes, 30 tRNA genes and 4 rRNA genes. Among them, 15 genes (*atpF*, *ndhA*, *ndhB*, *petB*, *petD*, *rpl2*, *rpl16*, *rpoC1*, *rps16*, *trnA-UGC*, *trnG-UCC*, *trnI-GAU*, *trnK-UUU*, *trnL-UAA* and *trnV-UAC*) have one intron, and three genes (*clpP*, *rps12* and *ycf3*) contain two introns. The overall GC content is 37.9%, and the corresponding values of the LSC, SSC, and IR regions are 35.9, 32.0, and 43.4%, respectively.

Phylogenetic analyses including *M. yangchunensis*, eight other Gentianales species and two outgroups of Lamiales, were performed using complete cp genomes. Sequences were aligned by MAFFT v7.017 plugin (Katoh et al. 2002) and visually checked and adjusted in Geneious. Phylogenetic analyses were carried out by RAxML v8.2 (Stamatakis 2014) using 1000 replicates of a rapid bootstrap analysis with GTRGAMMA substitution model. Phylogenetic analyses showed that *M. yangchunensis* is most closely related to *Mitrasacme pygmaea* (Figure 1), and similar to previous studies (Li et al. 2019), Gelsemiaceae and Loganiaceae form a single cluster with high bootstrap support (BS = 92%). Gentianaceae were resolved as sister to Gelsemiaceae + Loganiaceae with BS = 51%, and then clustered with Apocynaceae with BS = 100%. Besides, Rubiaceae is sister to the rest of the order with maximum support. The relationships between Gentianaceae and Gelsemiaceae + Loganiaceae were not well resolved, indicating that more taxa and nuclear genes of Gentianales should be sampled in future studies.

**Figure 1.**
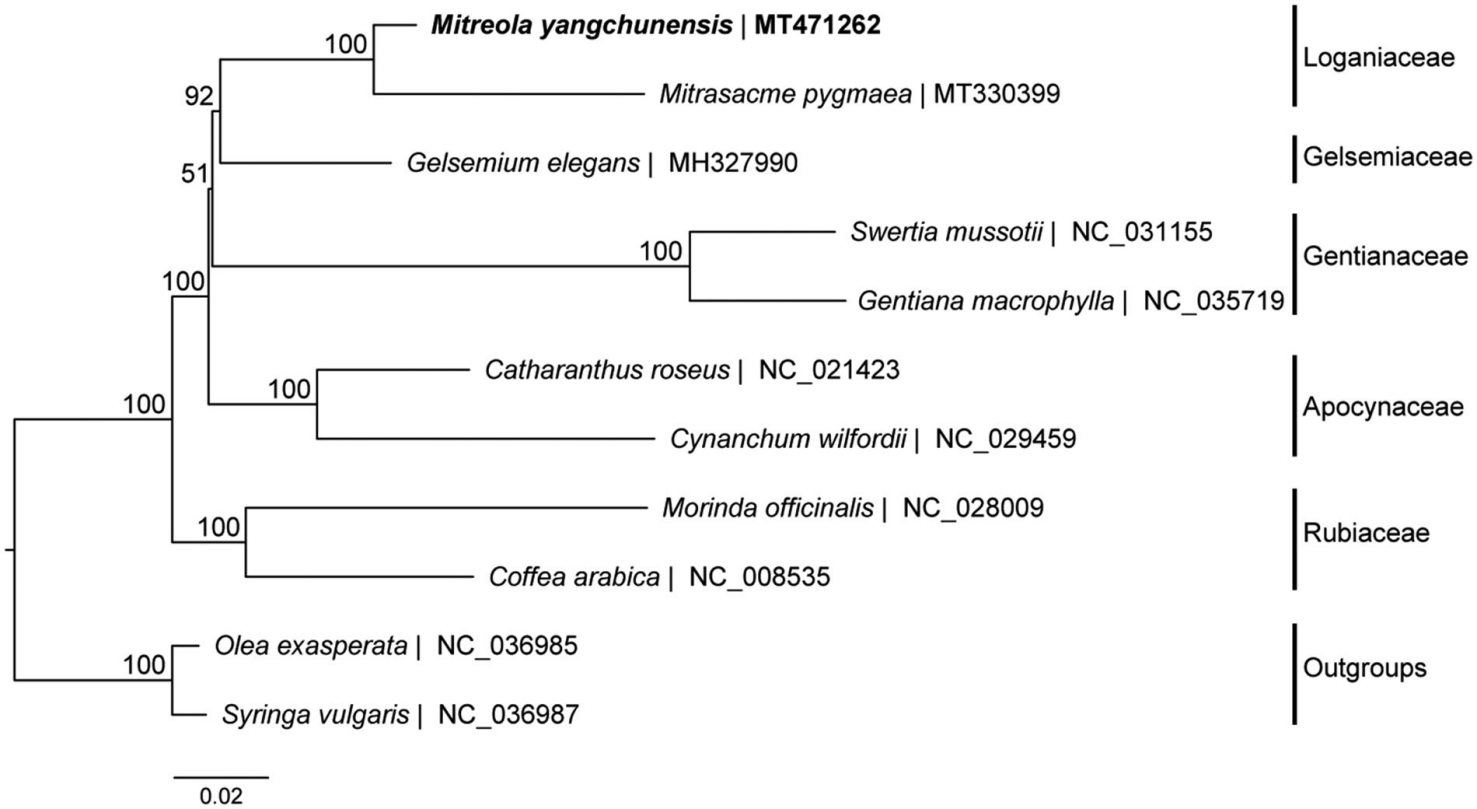
Maximum-likelihood phylogenetic tree based on complete cp genomes. Numbers close to each node are bootstrap support values.

## Data Availability

The data that support the findings of this study are openly available in GenBank at https://www.ncbi.nlm.nih.gov/genbank/, accession numbers [MT330399, MH327990, NC_008535, NC_021423, NC_028009, NC_029459, NC_031155, NC_035719, NC_036985, and NC_036987]. The complete chloroplast genome generated for this study has been deposited in GenBank with accession number MT471262. All high-throughput sequencing data files are available from the GenBank Sequence Read Archive (SRA) accession number: SRR12719804.
